# An update on national recommendations for the use of the adjuvanted recombinant zoster vaccine: Romy Weller

**DOI:** 10.1093/eurpub/ckac129.728

**Published:** 2022-10-25

**Authors:** R Parikh, R Widenmaier, R Weller, N Lecrenier

**Affiliations:** 1 Global Medical Affairs, GlaxoSmithKline, Wavre, Belgium; 2 Global Medical Affairs, GlaxoSmithKline, Rockville, USA; 3 Global Medical Affairs, GlaxoSmithKline, Munich, Germany

## Abstract

**Background:**

The adjuvanted recombinant zoster vaccine (RZV), first approved in 2017, has high, long-lasting efficacy against herpes zoster (HZ) and a clinically acceptable safety profile. In addition to the prevention of HZ in adults aged ≥50 years, the non-live RZV can be used from age 18 years in individuals with immunocompromised (IC) conditions. We reviewed the evolving landscape of national recommendations for RZV use.

**Methods:**

National health authority and vaccination committee websites of countries where RZV is approved were searched in March 2022.

**Results:**

Of 41 countries where RZV is licensed, 14 (Australia, Austria, Canada, Czech Republic, Germany, Ireland, Italy, Netherlands, New Zealand, Saudi Arabia, Spain, Switzerland, UK, US) provide national recommendations related to RZV; the majority are preferential to RZV or only recommend RZV. Overall, seven and seven countries recommend immunisation from age 50 years or 60/65 years, respectively. Of the seven countries that recommend immunisation from age 60/65 years, five recommend immunisation in individuals from age 50 years with comorbidities/IC conditions. Five countries recommend immunisation from age 18/19 years in individuals at increased risk of HZ due to immunosuppressive disease/treatment. In addition, six national recommendations refer to RZV safety and nine address prior HZ vaccination and/or infection. All recommendations outlined the RZV administration schedule.

**Conclusions:**

Although national recommendations can inform decision making in clinical practice, RZV recommendations are not available in all licensed countries. The recommendations highlight a trend in favour of the use of RZV for the prevention of HZ in older individuals and those with IC conditions.

Main messages: An increasing number of countries are providing recommendations for the use of RZV for the prevention of HZ in older individuals and those with IC conditions.

**Key messages:**

• An increasing number of countries are providing recommendations for the use of RZV for the prevention of HZ in older individuals.

• An increasing number of countries are providing recommendations for the use of RZV for the prevention of HZ in IC conditions.

